# Universal thermal climate index associations with mortality, hospital admissions, and road accidents in Bavaria

**DOI:** 10.1371/journal.pone.0259086

**Published:** 2021-11-17

**Authors:** Wael Ghada, Nicole Estrella, Donna P. Ankerst, Annette Menzel

**Affiliations:** 1 Department of Life Science Systems, Technical University of Munich, Freising, Germany; 2 Department of Mathematics, Technical University of Munich, Garching, Germany; 3 Institute for Advanced Study, Technical University of Munich, Garching, Germany; University of Vigo, SPAIN

## Abstract

When meteorological conditions deviate from the optimal range for human well-being, the risks of illness, injury, and death increase, and such impacts are feared in particular with more frequent and intense extreme weather conditions resulting from climate change. Thermal indices, such as the universal thermal climate index (UTCI), can better assess human weather-related stresses by integrating multiple weather components. This paper quantifies and compares the seasonal and spatial association of UTCI with mortality, morbidity, and road accidents in the federal state of Bavaria, Germany. Linear regression was applied to seasonally associate daily 56 million hospital admissions and 2.5 million death counts (1995–2015) as well as approximately 930,000 road accidents and 1.7 million people injured (2002–2015) with spatially interpolated same day- and lagged- (up to 14 days) average UTCI values. Additional linear regressions were performed stratifying by age, gender, region, and district. UTCI effects were clear in all three health outcomes studied: Increased UTCI resulted in immediate (1–2 days) rises in morbidity and even more strongly in mortality in summer, and lagged (up to 14 days) decreases in fall, winter, and spring. The strongest UTCI effects were found for road accidents where increasing UTCI led to immediate decreases in daily road accidents in winter but pronounced increases in all other seasons. Differences in UTCI effects were observed e.g. between in warmer north-western regions (Franconia, more districts with heat stress-related mortality, but hospital admissions for lung, heart and external reasons decreasing with summer heat stress), the touristic alpine regions in the south (immediate effect of increasing UTCI on road accidents in summer), and the colder south-eastern regions (increasing hospital admissions for lung, heart and external reasons in winter with UTCI). Districts with high percentages of elderly suffered from higher morbidity and mortality, particularly in winter. The influences of UTCI as well as the spatial and temporal patterns of this influence call for improved infrastructure planning and resource allocation in the health sector.

## Introduction

Climate change is altering the frequency, intensity, spatial extent, duration, and timing of extremes [1]. This affects morbidity, particularly among vulnerable populations [2], and extends to mortality and road accidents. Losses will intensify in absence of climate change adaptation and mitigation practices [3].

Among climate variables, air temperature remains the most studied predictor of morbidity. Higher temperatures were related to more emergency cases in summer [4], more nervous, circulatory and respiratory diseases [5], strokes [6], trauma [7–10], injuries [11, 12], and preterm births [13]. Heatwave days witnessed more emergency department admissions and higher mortality [14] due to heat strokes, sunstrokes, and fluid disorders [15]. Mortality rates increased on hot days [16], rapidly per degree above thresholds [17] and injury-related mortality increased with temperature in the US [18]. The reduction of mortality after heat-related mortality peaks, the so-called “harvesting effect”, disappeared in cases of extreme heat [16]. On the other hand, cold increased respiratory and circulatory diseases [5] as well as unintentional injuries [11] and led to high mortality levels in France [16]. Mortality due to cold spells was especially high among elderly, respiratory patients, and the less educated [19]. Consequently, temperature influences on morbidity and mortality differ between cold and warm seasons. More trauma patients, a higher proportion of young patients [20], and more orthopedic trauma consultation [10] occurred in summer than in winter in the US and the UK. Wider diurnal temperature ranges in cold seasons were associated with more patients with chronic respiratory diseases, but less in hot seasons [21]. Most interestingly exposure to heat in warm seasons had no impact on hospital admissions for cardiovascular and respiratory reasons in Spanish cities but was associated with higher mortality risks. In contrast, cold exposure was associated with more hospitalization, but lower mortality risks due to cardiovascular and respiratory reasons during cold seasons [22]. Thus, it is necessary to consider seasonal variations to understand the impact of weather conditions for health services [23]. In addition to temperature, other meteorological parameters for predicting morbidity and mortality are solar radiation, humidity, wind speed, precipitation, and foehn [4, 6, 8, 24, 25]. Following extreme weather events, there is an increased demand for emergency services and an increased mortality risk [2, 26]. Road accidents as well as associated injuries and fatalities have been influenced by both high and low temperatures [27–30], precipitation [31, 32], sunshine and wind speed [33], as well as by sandstorms [34]. Higher intensity of weather conditions increased road accidents [30, 35], particularly when temperatures were below freezing in Germany [36].

Thermal indices provide an excellent way to predict the demand for medical services due to adverse weather conditions more efficiently than direct weather variables [37, 38]. Indices, such as the Universal Thermal Climate Index (UTCI), have been recommended as physiologically relevant indices for biometeorology and climate impact studies [39]. UTCI better represented the physiological response of the human body, and was more sensitive to heat stress changes than other thermal indices [40]. Consequently, UTCI has been proposed as a basis for constructing heat warning systems [41], leading to assessments of its spatial and temporal variation [42–44]. An increase in UTCI has been observed for Europe over the last decade, with the south being more prone to heat stress than the north [45].

Various correlations between UTCI and morbidity/mortality have been addressed in recent studies. UTCI performed well in estimating the occupational heat stress in mines in Iran [46], the intensity of summer excess mortality in the Czech Republic [47], as well as in Europe, particularly in France during the heatwave of summer 2003 [45]. The impact of UTCI on mortality varied between warm and cold regions in Poland [48] and between rural and urban areas in the Czech Republic [37].

Within Germany, Bavaria is expected to suffer an increase in mortality rate due to climate change and population aging [49]. Temperature extremes induced higher mortality rates among the elderly [50] and higher cardiovascular mortality [51]. Also, ambulance activity in Munich was affected by temperature, humidity, sunshine, and precipitation [4], and severe trauma by foehn winds [25]. However, the influence of weather conditions on traffic accidents has not yet been studied at all. To the best of our knowledge, there have been no studies addressing and comparing the UTCI associations with mortality, morbidity, and traffic accidents.

We hypothesize that the influence of heat stress as assessed by UTCI on both morbidity and road accidents is comparable to the established influence of heat stress on mortality across seasons. We expect the spatial variation in these effects to be related to population characteristics. Therefore, the following questions are addressed (1) How do mortality, morbidity, and road accidents respond to variation in UTCI? (2) Does such an influence differ with seasons, age, sex, and regions within Bavaria?

By combining daily UTCI averages and daily counts of hospital admissions, death cases, and traffic accidents, this report quantifies the impact of UTCI on the population of Bavaria in order to mitigate severe impacts of weather extremes, improve the resilience and preparedness of health care systems, and reduce casualties.

## Materials and methods

### Morbidity, mortality, and road accidents

Bavarian hospital admissions and mortalities for the period 1995–2015 as well as road accidents for 2002–2015 were provided after anonymization by the Research Data Centers of the Federal Statistical Office and the Statistical Offices of the Federal States [52–54]. Access to this data and can be arranged through their website after strict procedures for ethical and privacy-related reasons. For further details https://www.forschungsdatenzentrum.de/en/request. Hospital admissions and mortality data comprised date, age, sex, and ICD-10 code, while the road accidents data included the number of people involved in each accident. A total of 56,028,368 hospital admissions, 2,557,651 mortalities, and 930,861 road accidents involving 1,753,980 injured persons were aggregated for this study (see Table 1). The three datasets may partially overlap; however, it is not possible to identify the overlapping cases. The daily counts of hospital admissions were itemized by sex, age (child: <18, adult: 18–70, senior: >70), diagnoses (heart, lungs, external), and death. Similarly, daily mortality counts were itemized by sex, age, and diagnoses. “External” here refers to injuries due to external causes such as falling, machinery, or fire.

**Table 1:** Total and average daily hospital admissions, mortality, and road accidents in Bavaria by season as well as by subgroups and regions.

VARIABLE	TOTAL COUNT	%	DAILY AVERAGE							
			Winter	Sd*	Spring	Sd	Summer	Sd	Fall	Sd
**Hospital admissions (1995–2015)**										
**Total**	**56028368**	**100**	**7019.1**	3143.5	**7501.8**	2878.6	**7301.6**	2578.8	**7392.4**	2670.6
**By subgroup**										
** Female**	**30062529**	**53.7**	**3752.2**	1629.1	**4040.4**	1496.5	**3903.9**	1327.6	**3978.9**	1383
** Male**	**25965546**	**46.3**	**3266.9**	1523.0	**3461.4**	1389.9	**3397.7**	1258.3	**3413.4**	1296.4
** Adult**	**32776409**	**58.5**	**4151.2**	1955.4	**4360.8**	1801.3	**4242.9**	1571.5	**4336.8**	1676.2
** Child**	**6092564**	**10.9**	**764.8**	237.3	**828.1**	219.7	**821.0**	207.1	**762.5**	187.2
** Senior**	**17158985**	**30.6**	**2103.1**	1055.2	**2312.9**	966.2	**2237.7**	895.8	**2293**	914.9
** Heart**	**1961849**	**3.5**	**248.3**	487.9	**268.9**	510.5	**244.2**	464.0	**261.7**	499.9
** Lungs**	**809849**	**1.4**	**117.3**	230.7	**115.6**	224.8	**91.8**	178.4	**97.8**	188.9
** External**	**1407912**	**2.5**	**171.8**	320.1	**182.9**	332	**197.6**	357.9	**181.7**	331.9
** Death**	**1187800**	**2.1**	**156.4**	51.7	**161.3**	38.1	**149.6**	33.9	**152.2**	33.3
**By Region**										
** Niederbayern**	**5168521**	**9.2**	**649.4**	273.6	**692.1**	244.8	**675.9**	218.0	**677.6**	226.8
** Oberbayern**	**19737933**	**35.2**	**2468.2**	1144.1	**2643.7**	1062.3	**2568.7**	957.0	**2611.3**	983.7
** Oberfranken**	**4789391**	**8.5**	**599.8**	268.2	**638.1**	244.4	**626.2**	219.3	**633.2**	228.4
** Oberpfalz**	**5127236**	**9.2**	**639.4**	285.7	**686.2**	261.8	**673.0**	235.5	**674.8**	242.0
** Schwaben**	**7174579**	**12.8**	**910.1**	386.0	**961.2**	347.0	**928.4**	310.5	**941.5**	322.3
** Unterfranken**	**6179137**	**11.0**	**772.5**	358.2	**826.4**	329.9	**806.0**	297.4	**817.1**	308.1
** Mittelfranken**	**7851571**	**14.0**	**979.6**	442.0	**1054.1**	403.4	**1023.4**	359.5	**1037**	373.3
**Mortality (1995–2015)**										
**Total**	**2557651**	**100**	**363.1**	33.9	**339.4**	33.6	**310.8**	26.3	**320.9**	24.4
**By subgroup**										
** Female**	**1356942**	**53.1**	**194.6**	21.5	**180.8**	21.1	**163.3**	16.4	**169.2**	15.6
** Male**	**1200709**	**46.9**	**168.5**	17.7	**158.6**	17.8	**147.5**	15.6	**151.7**	15.0
** Adult**	**964843**	**37.7**	**132.6**	17.1	**127.1**	15.9	**121.3**	15.2	**122.3**	14.5
** Child**	**16039**	**0.6**	**2.1**	1.6	**2.1**	1.6	**2.1**	1.5	**2.0**	1.5
** Senior**	**1576769**	**61.6**	**228.4**	29.6	**210.1**	30.1	**187.5**	23.9	**196.6**	23.3
** Heart**	**1151134**	**45.0**	**167.2**	21.7	**154.4**	19.4	**136**	15.5	**143.0**	16.6
** Lungs**	**171227**	**6.7**	**27.5**	8.2	**24.2**	7.6	**18.3**	4.9	**19.3**	5.3
** External**	**101565**	**4.0**	**12.7**	4.0	**13.2**	4.2	**13.9**	4.5	**13.0**	3.8
**By Region**										
** Niederbayern**	**250326**	**9.8**	**35.4**	6.8	**33.1**	6.3	**30.6**	6.0	**31.5**	6.0
** Oberbayern**	**799492**	**31.3**	**113.9**	13.6	**105.6**	13.3	**96.9**	11.5	**100.7**	11.4
** Oberfranken**	**261829**	**10.2**	**37.0**	6.9	**34.9**	6.6	**32.0**	5.9	**32.8**	5.8
** Oberpfalz**	**229686**	**9.0**	**32.5**	6.3	**30.6**	6.2	**28.0**	5.7	**28.7**	5.6
** Schwaben**	**370202**	**14.5**	**52.9**	8.2	**49.2**	8.2	**44.6**	7.1	**46.4**	7.2
** Unterfranken**	**276525**	**10.8**	**39.2**	7.3	**36.7**	7.0	**33.6**	6.4	**34.8**	6.3
** Mittelfranken**	**369591**	**14.5**	**52.3**	8.5	**49.3**	8.3	**45.2**	7.6	**46.1**	7.5
**Road accidents (2002–2015)**										
**Accidents**	**930861**	**100**	**152.4**	54.1	**174.1**	48.8	**213.0**	42.5	**188.1**	46.2
**Injured persons**	**1753980**	**100**	**286.4**	103.5	**330.2**	98.6	**395.3**	89.5	**359.3**	95.0

*Sd is the standard deviation of the daily count considering the number of days in each season.

**Fig 1:**
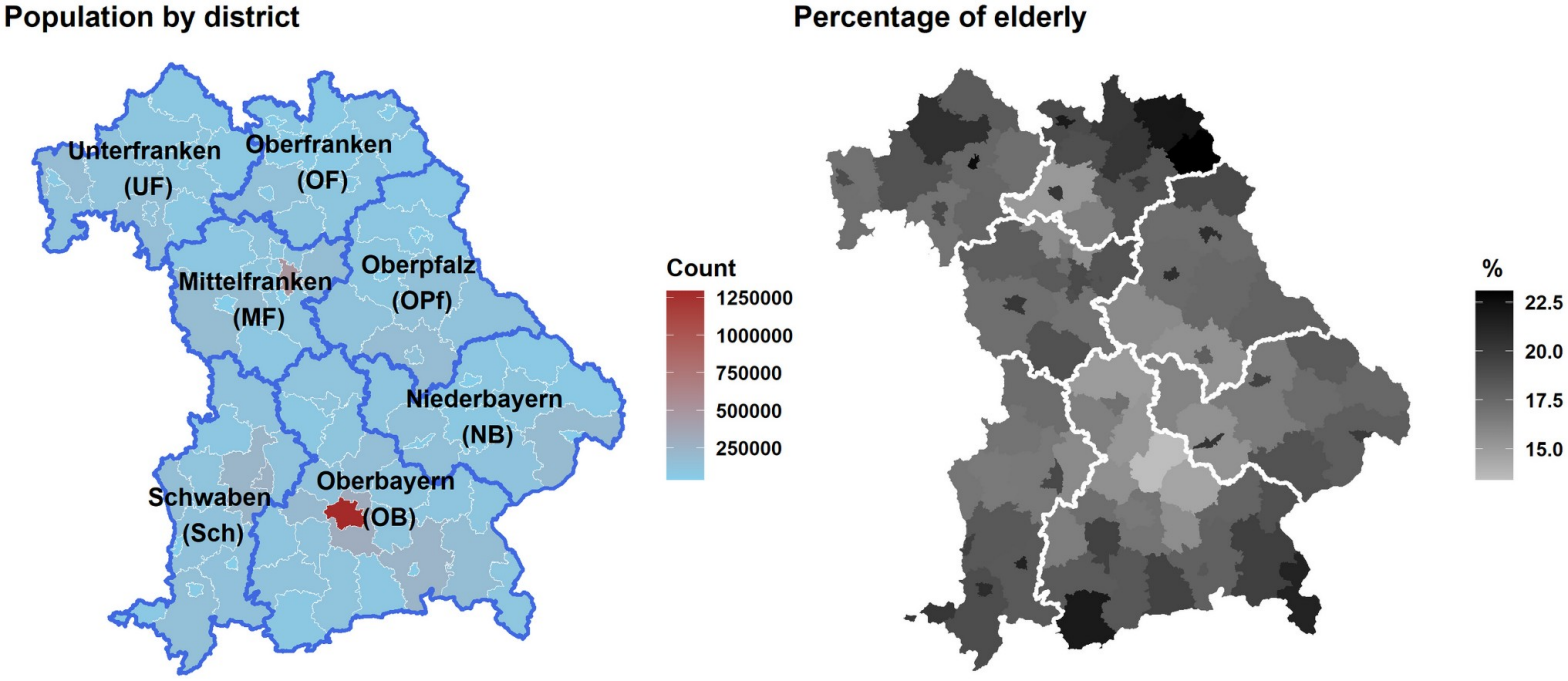
Population of Bavaria. The population in each district (left panel), and the percentage of elderly/senior (right panel). All values are averaged over the study period 1995–2015 [55]. Border shapefiles were provided with written permission by the Bundesamt für Kartographie und Geodäsie under the license (CC BY 4.0).

**Fig 2:**
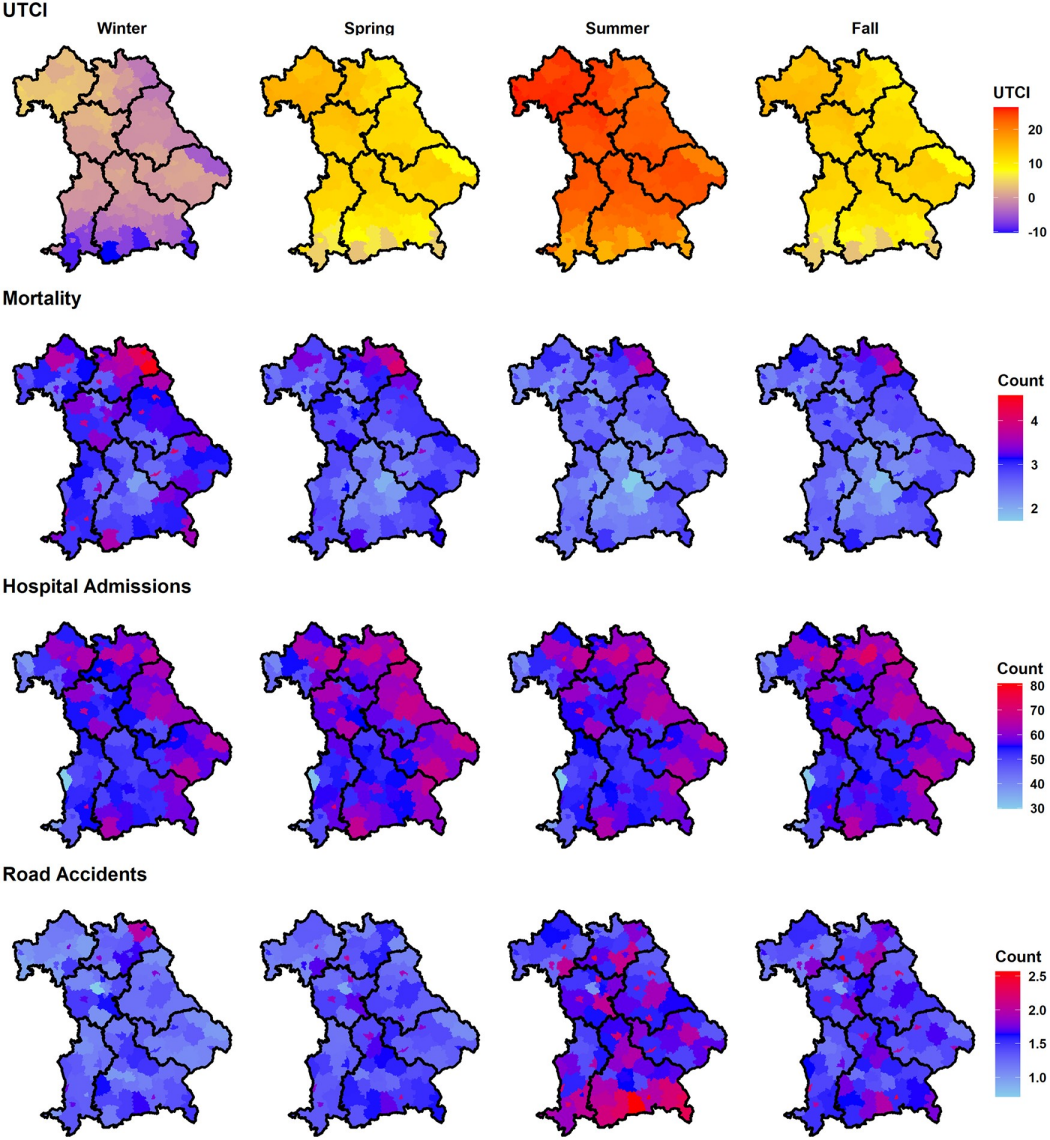
Summary of the data by season and region/district. The upper row of panels contains for each district the average UTCI value calculated for the corresponding season over the study period (1995–2015). The numbers of daily cases per 100,000 inhabitants are averaged for mortality (second row) and for hospital admissions (third row) over 1995–2015. The lower panel row represents the daily number of road accidents per 100,000 inhabitants averaged over 2002–2015. Border shapefiles were provided with written permission by the Bundesamt für Kartographie und Geodäsie under the license (CC BY 4.0).

It is important to note the spatial variation of population and age structure among Bavarian regions and districts (Fig 1 and S1 Fig). Therefore, the daily counts were calculated for the whole of Bavaria, for its seven regions, namely Lower-Bavaria/Niederbayern (NB), Upper-Bavaria/Oberbayern (OB), Upper-Franconia/Oberfranken (OF), Upper-Palatinate/Oberpfalz (OPf), Swabia/Schwaben (Sch), Lower-Franconia/Unterfranken (UF), Middle-Franconia/Mittelfranken (MF) (see S1 Table), and for its 96 districts (i.e. Landkreise) (Fig 2).

### UTCI

To determine the UTCI, the meteorological variables solar radiation, relative humidity, wind speed, and air temperature are required [56]. The German Meteorological Service (DWD) [57] provided hourly measurements for 44 stations throughout Bavaria between 1995–2015. To obtain precise UTCI values, detailed information on topography, human activities, and clothing in a physiological model is required. Since this information is not available, hourly UTCI values at individual stations were approximated by 6^th^ order polynomial regression functions [58]. Daily grids of UTCI at 200m resolution were then produced based on the daily averaged values of UTCI and integrated nested Laplace approximation (INLA) models [59]. From this analysis, mean daily UTCI values were extracted for Bavaria, each region, and district (Fig 2).

### Statistical methods

Multiple normal linear regression was used for association analyses of daily hospital admissions, death, and road accidents with UTCI, spatial and seasonal predictors. Lagged effects of UTCI up to 14 days were considered as potential predictors in a model for expected daily cases *E*(*Y*) (hospital admissions, deaths, or road accidents), which controlled for the year, official state holiday, and day of the week as shown below:

$$E\left(Y\right)=\alpha +\beta_{y}*y+\beta_{H}*H+\sum_{d=Monday}^{Saturday}\beta_{d}*d+\sum_{lag=0}^{lag=14}\beta_{UTCI_{lag}}*UTCI_{lag},$$ (1)

where α is the intercept, Y the year, β_y_ the change in E(Y) per year, H an indicator with value 1 for holidays and 0 otherwise, β_H_ the difference in counts between holidays and non-holidays, d the day of the week, β_d_ the difference in counts between day d and the reference day, UTCI_lag_ is the UTCI on lag days previously, and β_UTCI_lag_ is the change in counts for a unit increase in UTCI. Friday was chosen as the reference weekday for hospital admissions since weekdays at the start of the week may be more compromised / biased by planned surgeries or examinations. For road accidents, the choice was moved to Thursday since Mondays and Fridays witness different levels of transportation activity on the road due to weekend commuters and weekend activities. For mortality, Wednesday was chosen as the reference day at the middle of the weekdays, and to highlight the different situation compared to the weekends. Inclusion of the lagged UTCI effects as fixed effects in the model removed autocorrelation, justifying the assumption of independent errors as required for the models. Large sample sizes enabled the assumption of a Normal distribution by the central limit theorem, which was confirmed by residual Normal quantile plots.

Separate models were constructed for meteorological seasons (DJF winter, MAM spring, JJA summer, SON fall), for age, sex, and diagnosis subgroups, and for Bavaria, its seven regions, and 96 districts. The total number of analyses comprised 84 for Bavaria, 588 for regions, and 8064 for districts. Daily case numbers and UTCI varied between regions, districts, subgroups, and seasons (see also Fig 2), and hence were standardized by subtracting means and then dividing by the respective standard deviations (sd). Therefore, when interpreting the modelled effects for a target group, season, and spatial unit, the sd of the respective daily values of mortality, morbidity, and accidents has to be considered as the unit of change (see Table 1). For example, a *β*_*UTCI*_ value of 0.1 means that a change of one standard deviation in UTCI within a particular season and region (or district) was associated with a positive increase in the daily count of cases of the target group with a magnitude of 10% of its standard deviation in the respective season and region. Explanatory examples in each figure caption help with this calculation.

The set of predictors for each model was chosen by minimization of the Bayesian Information Criterion (BIC). Variance inflation factor values were mostly below 2 indicating low multicollinearity in the final models. All computations were performed in the R statistical software package (version 4.0.3) and all comparisons were tested at the two-sided 0.05 level of significance.

## Results

### Seasonal UTCI effects on daily morbidity, mortality, and road accidents

**Fig 3:**
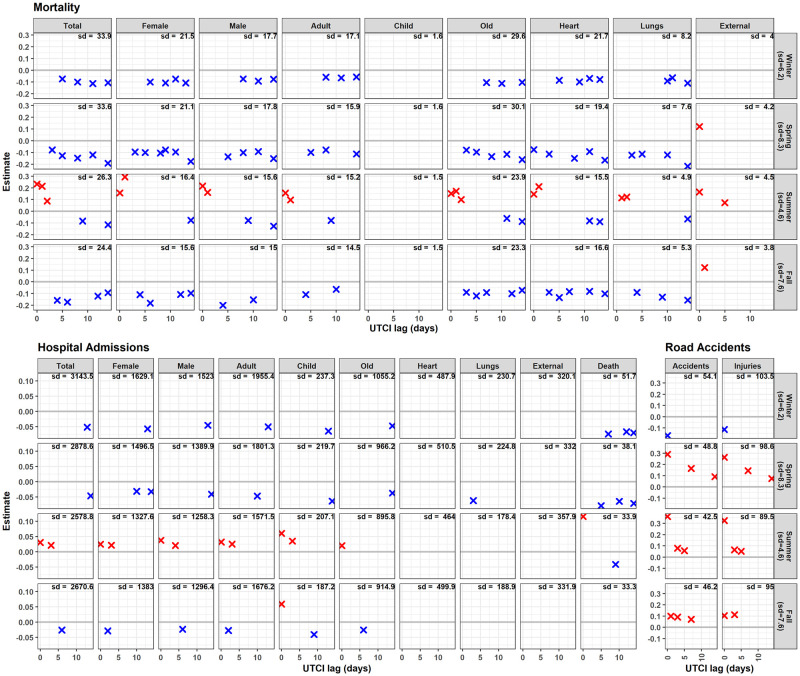
UTCI effects. UTCI effects on daily cases of mortality, hospital admissions, and road accidents for each subgroup and season in Bavaria. The horizontal axis represents the lag in days, and the vertical axis represents the effect estimate, either positive (red) or negative (blue). Crosses are lacking when the effect of UTCI was not significant for a particular lag in the corresponding model. Effect estimates are expressed in proportion of the standard deviation of daily number of cases within the corresponding subgroup (given in the upper right corner of each panel) when UTCI changes by one standard deviation of its daily value for the corresponding season. The numbers in the gray facet titles on the right are the standard deviations of UTCI within each season for the whole of Bavaria. The absence of the crosses indicates that the effect of UTCI was not significant for a particular lag in the corresponding model. Explanatory example: For winter, the UTCI sd is 6.2°C, the hospital admissions sd is 3143.5, and the estimated UTCI effect is -0.0513. This indicates a decrease in daily hospital admissions in Bavaria by -0.0513*3143.5 = 161 when the value of UTCI increases by 6.2°C.

In summer, higher UTCI was consistently associated with increasing daily numbers of mortality, hospital admissions, and road accidents (Fig 3). The strength of immediate UTCI effects (lag 0), however, differed substantially per 4.6°C UTCI increase: Daily hospital admissions increased by ~ +0.04 sd, mortality by ~ 0.20 sd, and road accidents by ~ +0.40 sd. Correspondingly, the category death within hospital admissions was characterized by larger UTCI effects (0.12 sd) than total admissions (0.04 sd).

In a few other cases amplifying (positive) UTCI effects were derived for seasons other than summer, namely for road accidents in spring and fall, children hospital admissions in fall as well as mortality due to external reasons in spring and fall. Here again, the UTCI effect size in spring was larger for road accidents than for external mortality.

Model results also pointed to temporal changes in UTCI effect based on the lag structure (Fig 3). In summer, an increase in mortality occurred up to three days after the increase in UTCI, but later there was a decrease within the second week. In the other seasons, however, no immediate effect was detected, but a decreasing effect of UTCI was apparent about three days after the UTCI change which lasted up to two weeks. Nevertheless, there were two exceptions: death among children was not influenced by UTCI fluctuations, and death due to external causes increased immediately after an increase in UTCI in spring, summer, and fall.

Higher summer UTCI values caused an immediate to three days lagged increase in Bavarian daily hospital admissions, but a decrease after one week in fall and after two weeks in winter and spring. The effect was consistent among sex and age groups, except for children’s hospital admissions, which increased immediately after an increase in UTCI in fall. No effect was detected on heart or external admission categories. However, lung-related admissions in spring decreased a couple of days after a UTCI increase. Remarkably, UTCI had an immediate positive effect on death within hospital admissions only in summer, and a negative lagged effect in winter, spring, and summer.

In winter, an increase in UTCI was associated with an immediate reduction in road accidents without any lagged effects. In other seasons, the association between UTCI and road accidents was always positive, very strong, and immediate in spring and summer and decreased over time up to one week in summer and fall, and up to two weeks in spring.

### Spatial variation of UTCI effects in summer and winter

**Fig 4:**
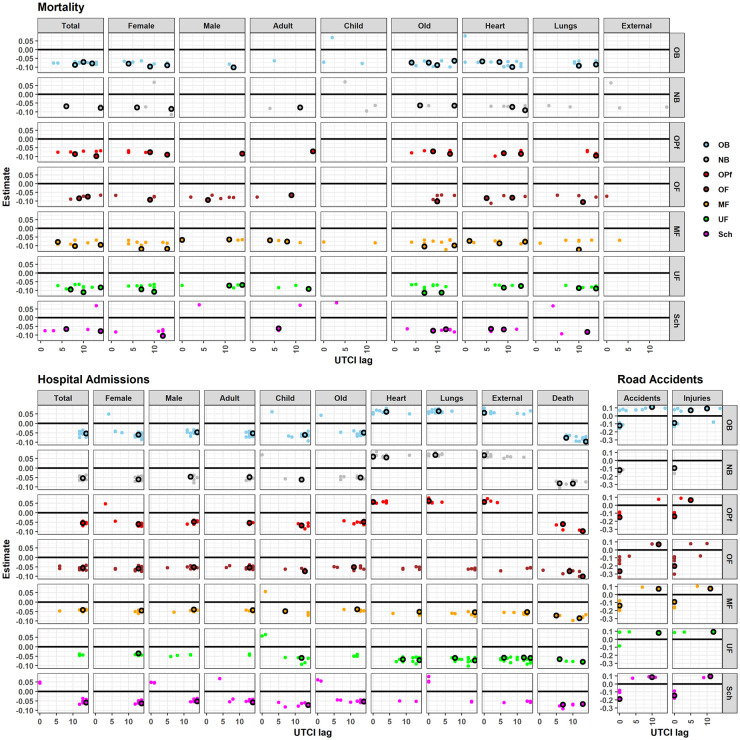
Winter UTCI effect. Winter UTCI effect on daily cases of mortality, hospital admissions, and road accidents for each subgroup and district. The horizontal axis represents the lag in days, and the vertical axis represents the effect estimate. It is expressed in proportion of the standard deviations of daily number of cases within the corresponding subgroup when UTCI changes by one standard deviation of its daily value. The black circles represent those effects for each region. The colored dots represent those effects for districts within the region. The absence of points indicates that the effect of UTCI was not significant for a particular lag in the corresponding model.

**Fig 5:**
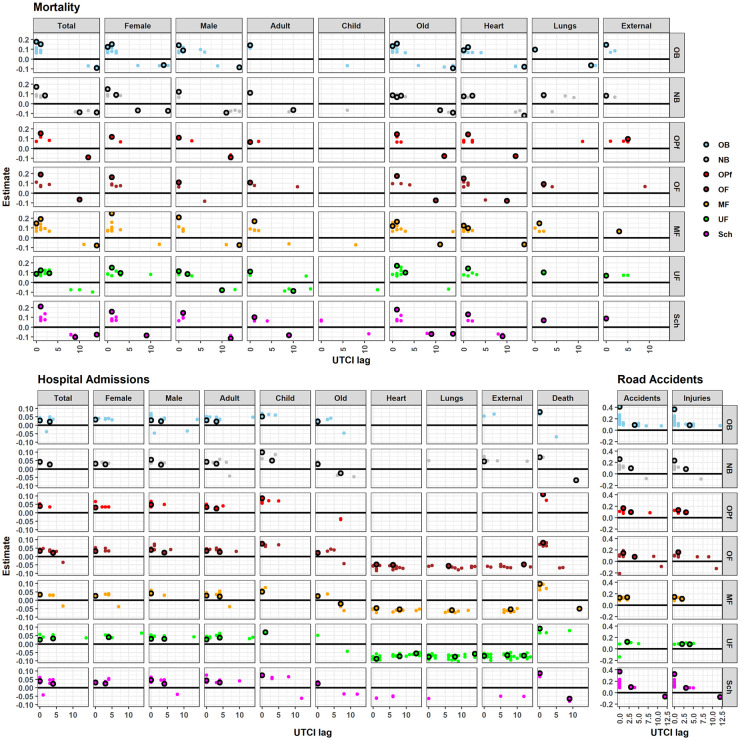
Summer UTCI effect. Summer UTCI effect on daily cases of mortality, hospital admissions, and road accidents for each subgroup and district. The horizontal axis represents the lag in days, and the vertical axis represents the effect estimate. It is expressed in proportion of the standard deviations of daily number of cases within the corresponding subgroup when UTCI changes by one standard deviation of its daily value. The black circles represent those effects for each region. The colored dots represent those effects for districts within the region. The absence of points indicates that the effect of UTCI was not significant for a particular lag in the corresponding model.

To examine the spatial variability of UTCI effects on the daily cases of morbidity, mortality, and road accidents, the effect estimates were compared between regions and districts. In order to tighten the results, we only discuss the summer and winter seasons in more detail (Figs 4 and 5), the corresponding results for spring and fall are in the supplement (S2 and S3 Figs).

In winter, there were no apparent differences in the UTCI effect structure on mortality among regions and their districts (Fig 4). Concerning morbidity, increases in UTCI were associated with immediate rises in hospital admissions due to heart, lungs, and external reasons in the three south-eastern (partly higher elevated and thus colder) regions of Bavaria, namely OB, NB, and OPf, but lagged reductions in MF and UF (i.e. the warmer regions of Bavaria) and some districts of the remaining regions. Additionally, increases in UTCI led to immediate reductions in road accidents in all regions except for UF, but delayed increases in all regions except NB and OPf.

Summerly UTCI effects on mortality were more frequent in two regions of Franconia (MF, UF), the relatively warmer north-western part of Bavaria, where more districts revealed significant UTCI effects (Fig 5). Quite strikingly, summer hospital admissions due to heart, lungs, and external causes were negatively correlated with UTCI change in OF, MF, and UF for 1 to 14 days lags structures. However, no effect was detected for all other regions, except NB where admissions due to external injuries were positively correlated with UTCI change. Additionally, deaths among hospital admissions decreased in the second week after UTCI increased in NB, MF, and Sch. Road accidents strongly increased with higher UTCI in summer in OB and Sch, the two main touristic regions in the south of Bavaria comprising alpine forelands and the alpine region.

For the two transitional seasons, spring and fall, some regional patterns in UTCI were apparent: Like in summer, the immediate increase in road accidents in spring with UTCI was high in OB, NB and Sch, which could be associated with the increase in deaths due to external causes in these three regions (S2 Fig). During fall, and despite the significant effect of UTCI on hospital admissions for all age groups for Bavaria, children’s hospital admissions were not affected by UTCI in MF and UF (S3 Fig). Similarly, the elderly were not affected in NB. In contrast, while UTCI had no effect on heart, lungs, or external hospital admissions, a significant negative effect appeared only in OF, MF, UF, and Sch for heart and lungs, and a positive effect for external cases in NB. The positive immediate effect of UTCI change on road accidents in Bavaria was limited to OB, NB, Sch, and reversed to a negative effect in OF and few other districts.

### Calendar effects

**Fig 6:**
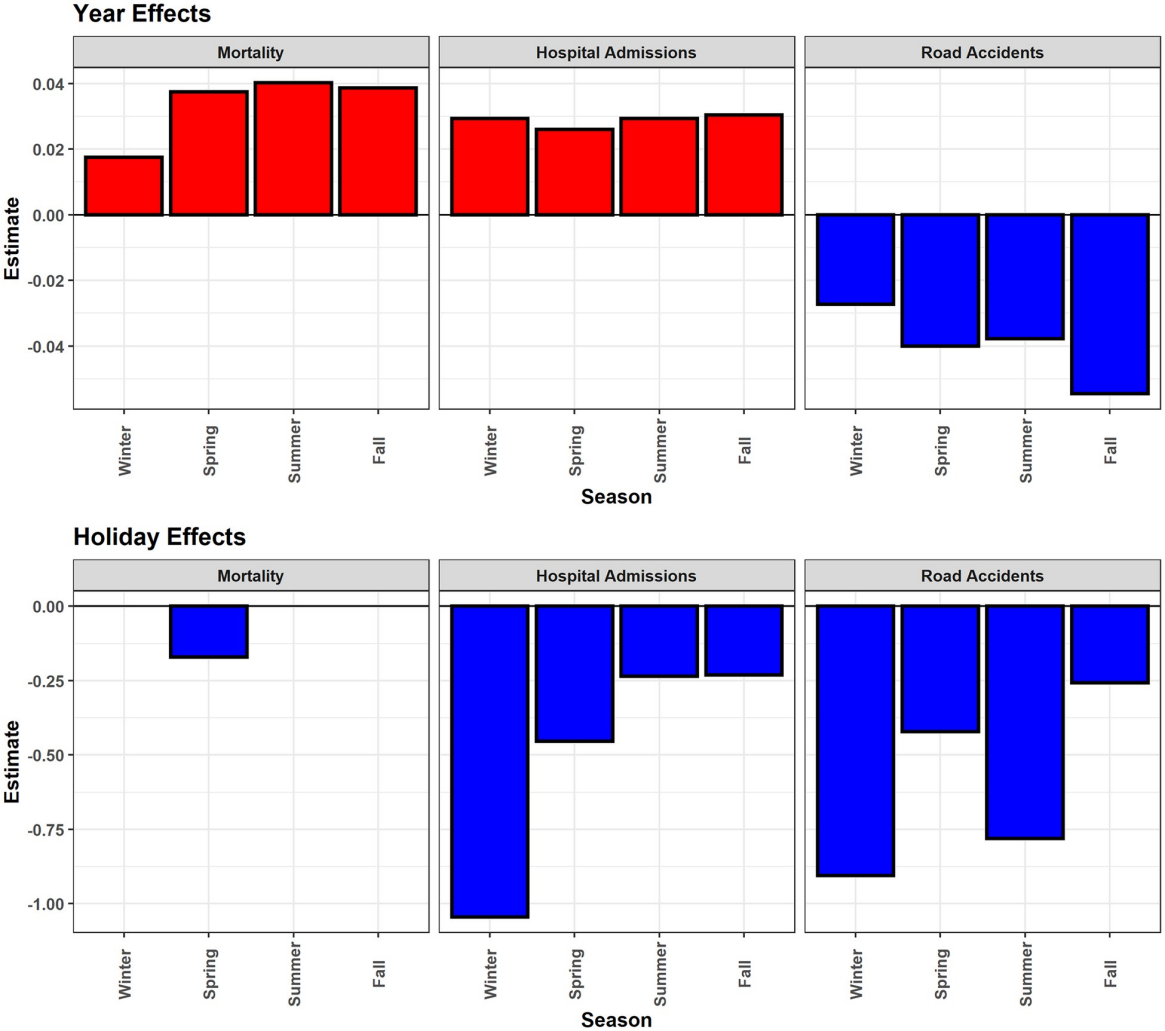
Year and holiday effects. Year and holiday effects on daily mortality, hospital admissions, and road accidents for each season, expressed as a proportion of the standard deviation of the respective daily number of cases within Bavaria. The absence of a column means that the effect of the year or holiday was not significant in the corresponding model. Explanatory example: For winter hospital admissions, the sd is 3143.5 (see Table 1), and the year effect for Bavaria is 0.02933. This indicates an increase in daily hospital admissions by 0.02933*3143.5 = 92.2 every year.

Daily hospital admissions and mortality increased across Bavaria during the 1995–2015 study period, while daily road accidents decreased (Fig 6). Overall, annual increases in daily hospital admissions ranged from 2.6% of sd in the spring to 3% in winter, corresponding to 75 and 92.2 additional daily cases per year, respectively. Total daily death counts in Bavaria increased during the study period, but not consistently across districts and for subgroups. Remarkably, the positive trend in mortality was the least in winter. While mortality increased for the elderly and for lungs cases, it decreased among adults, children, heart, and external cases (results for subgroups not shown). Daily road accidents and the resulting injuries decreased in all seasons, with the strongest decreases in fall, and the smallest decreases in winter.

Holidays significantly reduced the number of hospital admissions and road accidents but had no significant effect on deaths except in spring (Fig 6). Total daily hospital admissions were reduced especially in winter holidays (-105% of sd), followed by spring (-45% sd) and the reducing effect was smallest in summer and fall (-23% sd). The effect on children having school holidays was comparably high during summer (-42%), but lower in spring (-28%), and the least in fall (-9%). We observed only a marginal reduction of mortality during the spring holidays. Road accidents decreased during holidays by 90%, 78%, 42%, and 25% of the respective daily sds in winter, summer, spring, and fall.

**Fig 7:**
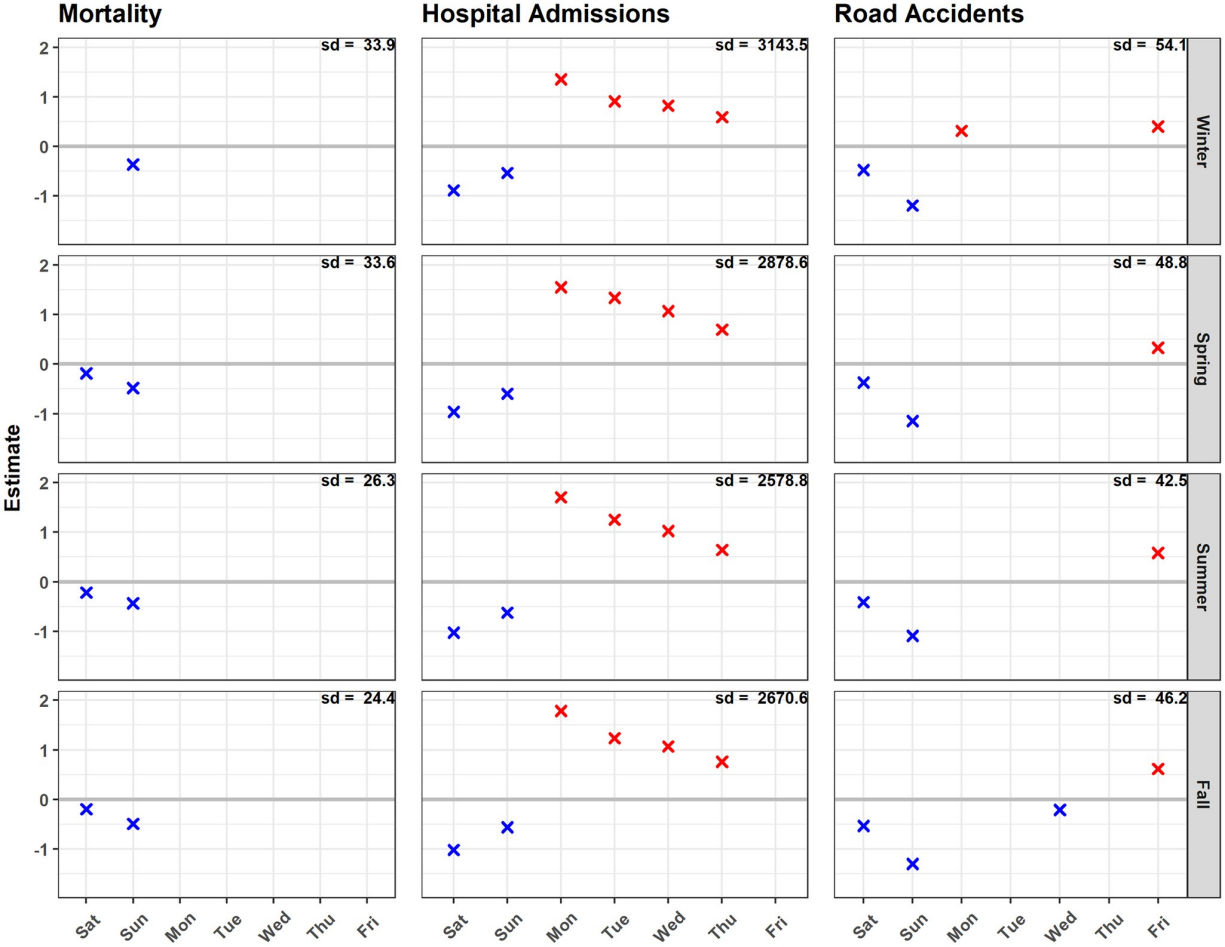
Day of the week effect. Day of the week effect on daily mortality, hospital admissions, and road accidents for each season, expressed in proportion of the standard deviations of daily number of cases within Bavaria compared to a reference day of the week (mortality Wednesday, hospital admissions Friday, road accidents Thursday). The color indicates the effect direction whether positive (red) or negative (blue). The number in the upper right corner of each panel indicates the standard deviation of daily cases in the relevant season for the whole of Bavaria. The absence of the cross means that the effect of the day was not significant in the corresponding model. Explanatory example: For winter hospital admissions, the sd is 3143.5, and the Saturday effect for Bavaria is -0.884. This indicates a decrease in daily hospital admissions by 0.884*3143.5 = 2779 on Saturdays compared to Fridays.

Hospital admissions were the highest on Mondays, and declined until Fridays, while Saturdays and Sundays witnessed the least daily cases (Fig 7). This pattern was spatially consistent, and across sex and age groups (results for subgroups not shown). The effect size was highest in summer and fall, and smallest in winter, slightly lower for children and the elderly, and mostly limited to Mondays and weekends for heart, lungs, external causes, and death within hospital admissions. Mortality in Bavaria generally dropped during the weekends, especially on Sundays. Children mortality was not affected by the day of the week, while death due to external injuries increased on Mondays. Road accidents increased on Fridays in all seasons, and on Mondays in winter. However, Sundays had the least number of accidents followed by Saturdays.

## Discussion

While few studies have separately examined the effects of weather on morbidity, mortality, and road accidents in Bavaria, this is the first study to compare the effects and consider the integrative effect of UTCI on these three public health outcomes. In addition, we examined variations of UTCI effects in space (districts, regions, and Bavaria), with season, and specific to different population groups. The results clearly indicated that UTCI had a significant effect on all three public health outcomes after adjusting for calendar effects.

### UTCI effect

The significant UTCI effect on hospital admissions, mortality, and road accidents in Bavaria extended to all regions, districts, and subgroups except children mortality which was not influenced by UTCI. This might be due to the low daily death cases among children, especially since an increase in children’s hospital and emergency admissions particularly in summer has been reported by other studies [4, 60].

Mortality and morbidity in Bavaria were similarly affected by UTCI as previously reported for temperatures, i.e. higher UTCI during summer caused an immediate increase in hospital admissions and mortality. The observed increase in hospital admissions agrees with the previous findings of higher ambulance activity in the Munich area with higher temperatures [4]. Similar increases in mortality with high UTCI have been reported for France [41], Czech Republic [47], and Bangladesh [61]. In fall, winter and spring, however, lower UTCI increased hospital admissions and mortality in Bavaria with a lag of one to two weeks. Such cold-related mortality has been equally reported for the Czech Republic [37] and Greece [62].

The unique result of our study is that for both promoting UTCI effects on mortality and morbidity in summer as well as mitigating UTCI effects in winter, the effects were clearly stronger for mortality than for morbidity by up to a factor of 5. Here, our study supports recent results for 52 Spanish cities [22] where heat exposure increased the risk of cardiovascular and respiratory mortality, but not hospital admissions.

The second important point of our study is a deeper look into the spatial patterns of such UTCI effects. Generally, the eastern districts of Bavaria have higher hospital admission rates per 100,000 inhabitants than the western ones (Fig 2), probably not due to more hospital beds (see S4 Fig for no apparent east-west differences), but likely due to a higher share of elderly people in the (north-) eastern districts of Bavaria, especially the districts of Wunsiedel and Hof, and in Garmisch-Partenkirchen in the south (Fig 1). It is well known that this senior part of the population is particularly vulnerable to heat/cold stress [49], probably resulting in higher hospital admissions and mortality in these districts at all times of the year, but especially in winter. These above-mentioned districts are characterized by lower UTCI values in winter, likely contributing to this spatial pattern in winter. A similar spatial variability of the UTCI effect on mortality has been recognized when comparing cool and warm cities in Poland [48].

Systematic reviews showed that heat-related morbidity and mortality differ by sex [63, 64]. Yet, no clear distinct differences between female and male vulnerability to UTCI were detected in this study.

Although there was a lack of a significant UTCI effect on heart, external, and lung hospital admissions for Bavaria, season-specific spatial variations became evident for these subgroups in summer and winter when considering the seven regions and the 96 districts, comparable to the study on cardiovascular- and respiratory-related hospital admissions in 52 Spanish cities [22]. For the (south-) eastern regions (OB, NB, and OPf), higher UTCI in summer had no effect on hospital admissions and lower UTCI in winter was associated with fewer hospital admissions. In contrast, for the (north-) western regions (OF, MF, and UF) higher UTCI in summer reduced hospital admissions and lower UTCI in winter was associated with increased hospital admissions. These east-west contrasts within Bavaria are striking since the UTCI heat effect on hospital admissions in summer was smaller in the comparably hotter regions (Fig 2) and in winter the UTCI cold effect was even reverse in the comparably cooler regions of Bavaria. Such region-specific variations in seasonal heat/cold stress effects on hospital admissions should be addressed when adjusting the best prevention and adaptation policies across the whole federal state of Bavaria accordingly.

Higher UTCI considerably increased road accidents in all seasons except for winter when more road accidents happen immediately after a decrease in UTCI. This can be well explained by bad driving conditions due to fog, precipitation, and the drop of temperatures below freezing level in winter, constituting major reasons for road accidents in Germany [36]. The increase in road accidents following high UTCI values in spring, summer, and fall coincide with a higher mortality (see Fig 3). This may be explained by a greater demand for leisure seeking activities and transportation. The immediate UTCI effect on road accidents in summer was about twice as strong as on mortality. Remarkably, during summer, road accidents increased in touristic districts especially in the alpine region in the south. The high number of road accidents in larger cities in all seasons was probably due to the high traffic density, which also peaks in summer with more non-local drivers. Since immediate amplifying UTCI effects were comparably strong in three seasons (spring through fall), awareness-raising activities and additional safety measures should target these regions before and during the peak periods.

### Confounding effects

All UTCI models accounted for other calendar-related effects (year, holidays, day of the week) on mortality, hospital admission, and road accidents. Since a deeper understanding of these confounding effects may support adaptation and policy measures, we will briefly discuss selected results in this respect.

The Bavarian population increased by 850,000 inhabitants over two decades, reaching 12.8 million in 2015 [65]. The corresponding increase in daily hospital admissions was concentrated in the elderly, children, and males, most likely due to a higher proportion of elderly in the German population being especially vulnerable to heat stress [49]. The increasing trend of death due to lung problems might be related to air pollution [66], as well as lung cancer and chronic obstructive pulmonary disease (COPD), especially among the increasing number of smoking women [67]. This highlights the importance of including the effect of air pollution in future studies, especially in the north-eastern districts, which are open to pollution sources from the east and commonly suffer blocking situations. Most Bavarian districts and regions had similar year effects on hospital admissions, mortality, and road accidents, with a few interesting exceptions. The districts with the highest population density, the cities of Munich, Nurnberg, and Augsburg, had a negative trend in total mortality, while most of the remaining districts had a positive trend, mirroring quite likely the concentration of the mobile, younger working population where the jobs are. Additionally, the observed discrepancies in mortality trends may be influenced by variations in birth rates, migration of young people to large cities, and investments in infrastructure. The observed reduction in daily road accidents over the study period could be associated with the technological improvement of safety measures [68], and the continuous efforts within the European Union to reduce casualties on the road [69]. There was only one district (Landshut) with a positive trend in road accidents in both summer and spring without any obvious explanation. Such deviations from the general patterns may be associated with the demographic characteristics, employment situation, number of hospital beds in these districts, and how they change over time. Factors like this should be addressed on the district level to resolve deficiencies and extract good policies on how to adapt to UTCI changes.

Holidays had almost no effect on mortality but witnessed fewer hospital admissions in all seasons, especially in winter, and for all regions, age, and sex groups. This evident winter reduction may be related to fewer outdoor activities. The reduction of mortality within hospital admissions during holidays was always less than the corresponding reduction in the total number of admissions. This finding may correspond to results of a meta-analysis reporting that the mortality rate is higher among patients admitted during holidays and weekends [70]. Seniors mostly do not depend on (school) holidays anymore, thus should not have holiday effects as e.g. shown by an absence of holiday effect on elderly emergency hospitalization in Munich [4]. However, in our study elderly hospital admissions did not deviate from the general pattern of the remaining age groups in Bavaria, and not even when considering the city of Munich. This discrepancy may be caused by the different years included in the emergency hospitalization study in Munich (2014–2018). The overall reducing effect of holidays was especially strong in winter and summer, the two seasons with a larger proportion of holidays in Bavaria.

Day of the week effects basically mirroring human behavior and habits were most prominent for hospital admissions, followed by road accidents, and least for mortality. Planned hospital admissions and a catch-up effect after the weekend perfectly explain the reduced hospital admissions on the weekend and the respective increase during weekdays, especially stronger at the beginning of the week. Weekends, especially Sundays had the least number of road accidents due to the reduced road activity in these days, whereas increased numbers on Mondays and Fridays may be related to weekly commuters. The excess in road accidents on Fridays, and Saturdays compared to Sundays might be due to alcohol consumption, risk-seeking, and leisure drives at night among young drivers [71].

### Future research and limitations

Our study confirmed that admission to hospitals, mortality, and road accidents were clearly associated with UTCI changes, with sizes of immediate effects in summer increasing in this order. Thus, the established influence of heat stress/UTCI extends to our health outcomes. In the next step, comparisons between prediction models which use thermal indices, particularly UTCI, and temperature should be carried out. In winter, the influence of precipitation might interfere with the UTCI influence. For example, it is evident that precipitation affects hospital admissions in the United States [24], while precipitation influences road accidents worldwide [32]. Particularly in the case of Munich, precipitation had a negative effect on emergency department visits in fall, and the number of female patients was negatively correlated with hail warnings and maximum precipitation intensity [4]. The simultaneous influence of precipitation and UTCI on mortality, morbidity, and road accidents is not addressed in this paper due to its complexity. However, this interaction calls for dedicated future research.

We expected UTCI effects to differ spatially within Bavaria and with subgroup characteristics. Therefore the specific patterns in the significant effects of UTCI on morbidity, mortality, and road accidents, that become obvious when conducting separate tests per season and age-gender subpopulation at different spatial scales, are extremely important. These results highlight the importance of planning climate change adaptation and mitigation efforts in both the local and regional context. According to literature, people who live in regions of moderate climate show higher sensitivity to weather extremes [72]. By applying this concept to Bavaria, inhabitants of warmer areas in Franconia and colder alpine regions are supposed to be less sensitive to UTCI variations in summer and winter, respectively. Therefore, our first, but not consistent results on spatial differences in UTCI effects should be intensified in future studies. Then, also the possibly amplified effect of UTCI in urban areas that might contribute to the increasing number of hospital admissions and mortality, especially among the elderly [61], should be addressed. Future research should also consider the possible influence of heat islands in large cities, and the moderating influence of vegetation and water bodies on the heat stress-related morbidity and mortality which has been recognized in other geographical locations [73–75].

The correlations reported between UTCI and the daily counts of Hospital admissions, death, and road accidents should be interpreted with care. Previous studies have demonstrated the causal connection between weather conditions, particularly heat-stress, and mortality in Bavarian large cities [50, 51]. Additionally, the observed correlations are highly influenced by the public behavior patterns. Specific ranges of UTCI at specific seasons may cause higher or lower levels of activity, use of transportation, interaction with the environment and other individuals, and stress which all eventually accumulate in causing the observed fluctuations in morbidity, mortality, and road accidents numbers. The available data does not contain enough details to attribute each case to a specific cause. However, our results indicate the surplus in cases which occur following UTCI fluctuations regardless of their nature of a direct or indirect link. This is particularly important for improving preparedness in the healthcare sector.

## Supporting information

S1 Fig Age structure of the population in Bavaria. The percentage of children and adults. All values are averaged over the study period 1995–2015 [55]. Border shapefiles were provided with written permission by the Bundesamt für Kartographie und Geodäsie under the license (CC BY 4.0).
(TIF)

S2 Fig Spring UTCI effect. Spring UTCI effect on the daily mortality, hospital admissions, and road accidents for each subgroup. The horizontal axis represents the lag in days, and the vertical axis represents the effect estimate. It is expressed in proportion of the standard deviations of daily number of cases within the corresponding subgroup when UTCI changes by one standard deviation of its daily value. The black circles represent those effects for each region. The colored dots represent those effects for districts within the region. The absence of the points means that the effect of UTCI was not significant for a particular lag in the corresponding model.
(TIF)

S3 Fig Fall UTCI effect. Fall UTCI effect on the daily mortality, hospital admissions, and road accidents for each subgroup. The horizontal axis represents the lag in days, and the vertical axis represents the effect estimate. It is expressed in proportion of the standard deviations of daily number of cases within the corresponding subgroup when UTCI changes by one standard deviation of its daily value. The black circles represent those effects for each region. The colored dots represent those effects for districts within the region. The absence of the points means that the effect of UTCI was not significant for a particular lag in the corresponding model.
(TIF)

S4 Fig Hospital beds in Bavaria. The number of hospital beds per 1000 inhabitants averaged over the study period 1995–2015 [76]. Border shapefiles were provided with written permission by the Bundesamt für Kartographie und Geodäsie under the license (CC BY 4.0).
(TIF)

S1 Table Percentages of hospital admissions, mortality, and road accidents in regions and by subcategories. (DOCX)

## References

[1] Seneviratne S, Nicholls N, Easterling D, Goodess C, Kanae S, Kossin J, et al. Changes in climate extremes and their impacts on the natural physical environment. 2012:109–230. doi: 10.7916/d8-6nbt-s431

[2] Sorensen CJ, Salas RN, Rublee C, Hill K, Bartlett ES, Charlton P, et al. Clinical Implications of Climate Change on US Emergency Medicine: Challenges and Opportunities. Ann Emerg Med. 2020; 76:168–78. doi: 10.1016/j.annemergmed.2020.03.010 32507491

[3] Ahmadalipour A, Moradkhani H. Escalating heat-stress mortality risk due to global warming in the Middle East and North Africa (MENA). Environment International. 2018; 117:215–25. doi: 10.1016/j.envint.2018.05.014 . 29763817

[4] Ghada W, Estrella N, Pfoerringer D, Kanz K-G, Bogner-Flatz V, Ankerst DP, et al. Effects of weather, air pollution and Oktoberfest on ambulance-transported emergency department admissions in Munich, Germany. Sci Total Environ. 2020:143772. doi: 10.1016/j.scitotenv.2020.143772 . 33229084

[5] Su X, Cheng Y, Wang Y, Liu Y, Li N, Li Y, et al. Regional Temperature-Sensitive Diseases and Attributable Fractions in China. Int J Environ Res Public Health. 2019; 17. doi: 10.3390/ijerph17010184 . 31888051 PMC6982219

[6] Sato T, Kusaka H, Hino H. Quantitative Assessment of the Contribution of Meteorological Variables to the Prediction of the Number of Heat Stroke Patients for Tokyo. SOLA. 2020; 16:104–8. doi: 10.2151/sola.2020-018

[7] Ali AM, Willett K. What is the effect of the weather on trauma workload? A systematic review of the literature. Injury. 2015; 46:945–53. doi: 10.1016/j.injury.2015.03.016 . 25816705

[8] Bundi M, Meier L, Amsler F, Gross T. Wie hängen Eintreffen und Outcome schwerer Verletzter im Traumazentrum von Wetter, Tages- und Jahreszeit ab. Unfallchirurg. 2018; 121:10–9. doi: 10.1007/s00113-016-0267-0 . 27778061

[9] Watson KE, Gardiner KM, Singleton JA. The impact of extreme heat events on hospital admissions to the Royal Hobart Hospital. J Public Health (Oxf). 2020; 42:333–9. doi: 10.1093/pubmed/fdz033 . 31220305

[10] Wilson JM, Staley CA, Boden AL, Boissonneault AR, Schwartz AM, Schenker ML. The Effect of Season and Weather on Orthopaedic Trauma: Consult Volume Is Significantly Correlated with Daily Weather. Adv Orthop. 2018; 2018:6057357. doi: 10.1155/2018/6057357 . 30245890 PMC6139230

[11] Im Otte Kampe E, Kovats S, Hajat S. Impact of high ambient temperature on unintentional injuries in high-income countries: a narrative systematic literature review. BMJ Open. 2016; 6:e010399. doi: 10.1136/bmjopen-2015-010399 . 26868947 PMC4762150

[12] Lee H, Myung W, Kim H, Lee E-M, Kim H. Association between ambient temperature and injury by intentions and mechanisms: A case-crossover design with a distributed lag nonlinear model. Sci Total Environ. 2020; 746:141261. doi: 10.1016/j.scitotenv.2020.141261 . 32745866

[13] Ilango SD, Weaver M, Sheridan P, Schwarz L, Clemesha RES, Bruckner T, et al. Extreme heat episodes and risk of preterm birth in California, 2005–2013. Environment International. 2020; 137:105541. doi: 10.1016/j.envint.2020.105541 . 32059147

[14] Williams S, Nitschke M, Weinstein P, Pisaniello DL, Parton KA, Bi P. The impact of summer temperatures and heatwaves on mortality and morbidity in Perth, Australia 1994–2008. Environment International. 2012; 40:33–8. doi: 10.1016/j.envint.2011.11.011 . 22280925

[15] Hopp S, Dominici F, Bobb JF. Medical diagnoses of heat wave-related hospital admissions in older adults. Prev Med. 2018; 110:81–5. doi: 10.1016/j.ypmed.2018.02.001 . 29428173 PMC6040588

[16] Pascal M, Wagner V, Corso M, Laaidi K, Ung A, Beaudeau P. Heat and cold related-mortality in 18 French cities. Environment International. 2018; 121:189–98. doi: 10.1016/j.envint.2018.08.049 . 30216771

[17] Calleja-Agius J, England K, Calleja N. The effect of global warming on mortality. Early Hum Dev. 2020:105222. doi: 10.1016/j.earlhumdev.2020.105222 . 33097356 PMC7536190

[18] Merrill RM. Injury-Related Deaths according to Environmental, Demographic, and Lifestyle Factors. J Environ Public Health. 2019; 2019:6942787. doi: 10.1155/2019/6942787 . 30944571 PMC6421738

[19] Chen J, Yang J, Zhou M, Yin P, Wang B, Liu J, et al. Cold spell and mortality in 31 Chinese capital cities: Definitions, vulnerability and implications. Environment International. 2019; 128:271–8. doi: 10.1016/j.envint.2019.04.049 . 31071590

[20] Hind J, Lahart IM, Jayakumar N, Athar S, Fazal MA, Ashwood N. Seasonal variation in trauma admissions to a level III trauma unit over 10 years. Injury. 2020; 51:2209–18. doi: 10.1016/j.injury.2020.07.014 . 32703642

[21] Wang Z, Zhou Y, Luo M, Yang H, Xiao S, Huang X, et al. Association of diurnal temperature range with daily hospitalization for exacerbation of chronic respiratory diseases in 21 cities, China. Respir Res. 2020; 21:251. doi: 10.1186/s12931-020-01517-7 . 32993679 PMC7526384

[22] Iñiguez C, Royé D, Tobías A. Contrasting patterns of temperature related mortality and hospitalization by cardiovascular and respiratory diseases in 52 Spanish cities. Environ Res. 2020; 192:110191. doi: 10.1016/j.envres.2020.110191 . 32980302

[23] Peña‐Angulo D, Reig‐Gracia F, Domínguez‐Castro F, Revuelto J, Aguilar E, Schrier G, et al. ECTACI: European Climatology and Trend Atlas of Climate Indices (1979–2017). J Geophys Res Atmos. 2020; 125. doi: 10.1029/2020JD032798

[24] Bobb JF, Ho KKL, Yeh RW, Harrington L, Zai A, Liao KP, et al. Time-Course of Cause-Specific Hospital Admissions During Snowstorms: An Analysis of Electronic Medical Records From Major Hospitals in Boston, Massachusetts. Am J Epidemiol. 2017; 185:283–94. doi: 10.1093/aje/kww219 . 28137774 PMC5860478

[25] Greve F, Kanz K-G, Zyskowski M, Matthey F von, Biberthaler P, Muthers S, et al. The influence of foehn winds on the incidence of severe injuries in southern Bavaria—an analysis of the TraumaRegister DGU^®^. BMC Musculoskelet Disord. 2020; 21:568. doi: 10.1186/s12891-020-03572-z . 32825813 PMC7442979

[26] Lane K, Charles-Guzman K, Wheeler K, Abid Z, Graber N, Matte T. Health effects of coastal storms and flooding in urban areas: a review and vulnerability assessment. J Environ Public Health. 2013; 2013:913064. doi: 10.1155/2013/913064 . 23818911 PMC3683478

[27] Zhan Z-Y, Yu Y-M, Chen T-T, Xu L-J, Ou C-Q. Effects of hourly precipitation and temperature on road traffic casualties in Shenzhen, China (2010–2016): A time-stratified case-crossover study. Sci Total Environ. 2020; 720:137482. doi: 10.1016/j.scitotenv.2020.137482 . 32145618

[28] Drosu A, Cofaru C, Popescu MV. Influence of Weather Conditions on Fatal Road Accidents on Highways and Urban and Rural Roads in Romania. Int.J Automot Technol. 2020; 21:309–17. doi: 10.1007/s12239-020-0029-4

[29] Wu CYH, Zaitchik BF, Gohlke JM. Heat waves and fatal traffic crashes in the continental United States. Accid Anal Prev. 2018; 119:195–201. doi: 10.1016/j.aap.2018.07.025 . 30048841 PMC6675573

[30] Lee W-K, Lee H-A, Hwang S, Kim H, Lim Y-H, Hong Y-C, et al. A time series study on the effects of cold temperature on road traffic injuries in Seoul, Korea. Environ Res. 2014; 132:290–6. doi: 10.1016/j.envres.2014.04.019 . 24834824

[31] Liu A, Soneja SI, Jiang C, Huang C, Kerns T, Beck K, et al. Frequency of extreme weather events and increased risk of motor vehicle collision in Maryland. Sci Total Environ. 2017; 580:550–5. doi: 10.1016/j.scitotenv.2016.11.211 . 27988189

[32] Theofilatos A, Yannis G. A review of the effect of traffic and weather characteristics on road safety. Accid Anal Prev. 2014; 72:244–56. doi: 10.1016/j.aap.2014.06.017 . 25086442

[33] Gao J, Chen X, Woodward A, Liu X, Wu H, Lu Y, et al. The association between meteorological factors and road traffic injuries: a case analysis from Shantou city, China. Sci Rep. 2016; 6:37300. doi: 10.1038/srep37300 . 27853316 PMC5112526

[34] Islam MM, Alharthi M, Alam MM. The Impacts of Climate Change on Road Traffic Accidents in Saudi Arabia. Climate. 2019; 7:103. doi: 10.3390/cli7090103

[35] Bergel-Hayat R, Debbarh M, Antoniou C, Yannis G. Explaining the road accident risk: weather effects. Accid Anal Prev. 2013; 60:456–65. doi: 10.1016/j.aap.2013.03.006 . 23928504

[36] Becker N, Rust HW, Ulbrich U. Predictive modeling of hourly probabilities for weather-related road accidents. Nat Hazards Earth Syst Sci. 2020; 20:2857–71. doi: 10.5194/nhess-20-2857-2020

[37] Urban A, Kyselý J. Comparison of UTCI with other thermal indices in the assessment of heat and cold effects on cardiovascular mortality in the Czech Republic. Int J Environ Res Public Health. 2014; 11:952–67. doi: 10.3390/ijerph110100952 . 24413706 PMC3924484

[38] Pan R, Gao J, Wang X, Bai L, Wei Q, Yi W, et al. Impacts of exposure to humidex on the risk of childhood asthma hospitalizations in Hefei, China: Effect modification by gender and age. Sci Total Environ. 2019; 691:296–305. doi: 10.1016/j.scitotenv.2019.07.026 . 31323575

[39] Jendritzky G, Dear R de, Havenith G. UTCI—why another thermal index. Int J Biometeorol. 2012; 56:421–8. doi: 10.1007/s00484-011-0513-7 . 22187087

[40] Blazejczyk K, Epstein Y, Jendritzky G, Staiger H, Tinz B. Comparison of UTCI to selected thermal indices. Int J Biometeorol. 2012; 56:515–35. doi: 10.1007/s00484-011-0453-2 . 21614619 PMC3337419

[41] Di Napoli C, Pappenberger F, Cloke HL. Verification of Heat Stress Thresholds for a Health-Based Heat-Wave Definition. Journal of Applied Meteorology and Climatology. 2019; 58:1177–94. doi: 10.1175/JAMC-D-18-0246.1

[42] Tomczyk AM, Owczarek M. Occurrence of strong and very strong heat stress in Poland and its circulation conditions. Theor Appl Climatol. 2020; 139:893–905. doi: 10.1007/s00704-019-02998-3

[43] Wu F, Yang X, Shen Z. Regional and seasonal variations of outdoor thermal comfort in China from 1966 to 2016. Sci Total Environ. 2019; 665:1003–16. doi: 10.1016/j.scitotenv.2019.02.190 . 30893732

[44] Vinogradova V. Using the Universal Thermal Climate Index (UTCI) for the assessment of bioclimatic conditions in Russia. Int J Biometeorol. 2020:1–11. doi: 10.1007/s00484-020-01901-4 . 32383024

[45] Di Napoli C, Pappenberger F, Cloke HL. Assessing heat-related health risk in Europe via the Universal Thermal Climate Index (UTCI). Int J Biometeorol. 2018; 62:1155–65. doi: 10.1007/s00484-018-1518-2 . 29546489 PMC6028891

[46] Nassiri P, Monazzam MR, Golbabaei F, Dehghan SF, Rafieepour A, Mortezapour AR, et al. Application of Universal Thermal Climate Index (UTCI) for assessment of occupational heat stress in open-pit mines. Industrial health. 2017; 55:437–43. doi: 10.2486/indhealth.2017-0018 . 28804096 PMC5633359

[47] Urban A, Hondula DM, Hanzlíková H, Kyselý J. The predictability of heat-related mortality in Prague, Czech Republic, during summer 2015-a comparison of selected thermal indices. Int J Biometeorol. 2019; 63:535–48. doi: 10.1007/s00484-019-01684-3 . 30739159

[48] Kuchcik M. Mortality and thermal environment (UTCI) in Poland-long-term, multi-city study. Int J Biometeorol. 2020:1–13. doi: 10.1007/s00484-020-01995-w . 32880062 PMC8370924

[49] Rai M, Breitner S, Wolf K, Peters A, Schneider A, Chen K. Impact of climate and population change on temperature-related mortality burden in Bavaria, Germany. Environ Res Lett. 2019; 14:124080. doi: 10.1088/1748-9326/ab5ca6

[50] Breitner S, Wolf K, Devlin RB, Diaz-Sanchez D, Peters A, Schneider A. Short-term effects of air temperature on mortality and effect modification by air pollution in three cities of Bavaria, Germany: a time-series analysis. Sci Total Environ. 2014; 485–486:49–61. doi: 10.1016/j.scitotenv.2014.03.048 . 24704956

[51] Breitner S, Wolf K, Peters A, Schneider A. Short-term effects of air temperature on cause-specific cardiovascular mortality in Bavaria, Germany. Heart. 2014; 100:1272–80. doi: 10.1136/heartjnl-2014-305578 . 24906508

[52] Research Data Center of the Federal Statistical Office and the Statistical Offices of the federal states (RDC). Krankenhausstatistik Teil II: Diagnosen der Krankenhauspatienten (23131).; 1995–2015.

[53] Research Data Center of the Federal Statistical Office and the Statistical Offices of the federal states (RDC). Todesursachenstatistik (23211).; 1995–2015.

[54] Research Data Center of the Federal Statistical Office and the Statistical Offices of the federal states (RDC). Statistik der Straßenverkehrsunfälle (46241).; 2002–2015.

[55] Federal Statistical Office Germany. Population: Administrative districts, reference date. Table: 12411–0015. © Statistisches Bundesamt (Destatis) [updated 25 Jan 2021; cited 25 Jan 2021]. https://www-genesis.destatis.de/genesis//online?operation=table&code=12411-0015#astructure.

[56] Błażejczyk K, Jendritzky G, Bröde P, Fiala D, Havenith G, Epstein Y, et al. An introduction to the Universal Thermal Climate Index (UTCI). Geogr Pol. 2013; 86:5–10. doi: 10.7163/GPol.2013.1

[57] Deutscher Wetterdienst. Hourly Climate Observations, Germany [updated 10 Dec 2020; cited 10 Dec 2020]. ftp://opendata.dwd.de/climate_environment/CDC/observations_germany/climate/hourly/.

[58] Bröde P, Fiala D, Błażejczyk K, Holmér I, Jendritzky G, Kampmann B, et al. Deriving the operational procedure for the Universal Thermal Climate Index (UTCI). Int J Biometeorol. 2012; 56:481–94. doi: 10.1007/s00484-011-0454-1 . 21626294

[59] Blangiardo M, Cameletti M, Baio G, Rue H. Spatial and spatio-temporal models with R-INLA. Spatial and Spatio-temporal Epidemiology. 2013; 4:33–49. Epub 2013/01/02. doi: 10.1016/j.sste.2012.12.001 . 23481252

[60] Winquist A, Grundstein A, Chang HH, Hess J, Sarnat SE. Warm season temperatures and emergency department visits in Atlanta, Georgia. Environ Res. 2016; 147:314–23. Epub 2016/02/27. doi: 10.1016/j.envres.2016.02.022 . 26922412 PMC4821766

[61] Burkart K, Breitner S, Schneider A, Khan MMH, Krämer A, Endlicher W. An analysis of heat effects in different subpopulations of Bangladesh. Int J Biometeorol. 2014; 58:227–37. Epub 2013/05/21. doi: 10.1007/s00484-013-0668-5 . 23689928

[62] Nastos PT, Matzarakis A. The effect of air temperature and human thermal indices on mortality in Athens, Greece. Theor Appl Climatol. 2012; 108:591–9. doi: 10.1007/s00704-011-0555-0

[63] Gifford RM, Todisco T, Stacey M, Fujisawa T, Allerhand M, Woods DR, et al. Risk of heat illness in men and women: A systematic review and meta-analysis. Environ Res. 2019; 171:24–35. Epub 2018/10/25. doi: 10.1016/j.envres.2018.10.020 . 30641370

[64] van Steen Y, Ntarladima A-M, Grobbee R, Karssenberg D, Vaartjes I. Sex differences in mortality after heat waves: are elderly women at higher risk. Int Arch Occup Environ Health. 2019; 92:37–48. Epub 2018/10/06. doi: 10.1007/s00420-018-1360-1 . 30293089

[65] Federal Statistical Office Germany. Bevölkerung: Gemeinde, Geschlecht, Quartale, Jahre, table 12411-009z. © Statistisches Bundesamt (Destatis) [updated 22 Jan 2021; cited 22 Jan 2021]. https://www.statistikdaten.bayern.de/genesis//online/data?operation=table&code=12411-009z.

[66] Liu C, Chen R, Sera F, Vicedo-Cabrera AM, Guo Y, Tong S, et al. Ambient Particulate Air Pollution and Daily Mortality in 652 Cities. N Engl J Med. 2019; 381:705–15. doi: 10.1056/NEJMoa1817364 . 31433918 PMC7891185

[67] RKI. Time trends in incidence and mortality of respiratory diseases of high public health relevance in Germany. RKI-Bib1 (Robert Koch-Institut); 2017. 10.17886/RKI-GBE-2017-061 PMC10165912 37168954

[68] TraumaRegister DGU. 20 years of trauma documentation in Germany—actual trends and developments. Injury. 2014; 45 Suppl 3:S14–9. doi: 10.1016/j.injury.2014.08.012 . 25284227

[69] European Commission. Roadmap to a single European transport area. towards a competitive and resource efficient transport system COM(2011) 144 final. White paper. Luxembourg: Publications Office of the European Union 2011 [updated 18 Jan 2021; cited 18 Jan 2021]. https://eur-lex.europa.eu/legal-content/EN/TXT/?uri=CELEX:52011DC0144.

[70] Pauls LA, Johnson-Paben R, McGready J, Murphy JD, Pronovost PJ, Wu CL. The Weekend Effect in Hospitalized Patients: A Meta-Analysis. J Hosp Med. 2017; 12:760–6. doi: 10.12788/jhm.2815 . 28914284

[71] Scheiner J, Holz-Rau C. A residential location approach to traffic safety: two case studies from Germany. Accid Anal Prev. 2011; 43:307–22. Epub 2010/10/08. doi: 10.1016/j.aap.2010.08.029 . 21094329

[72] Guo Y, Gasparrini A, Armstrong BG, Tawatsupa B, Tobias A, Lavigne E, et al. Heat Wave and Mortality: A Multicountry, Multicommunity Study. Environ Health Perspect. 2017; 125:87006. Epub 2017/08/10. doi: 10.1289/EHP1026 . 28886602 PMC5783630

[73] Burkart K, Meier F, Schneider A, Breitner S, Canário P, Alcoforado MJ, et al. Modification of Heat-Related Mortality in an Elderly Urban Population by Vegetation (Urban Green) and Proximity to Water (Urban Blue): Evidence from Lisbon, Portugal. Environ Health Perspect. 2016; 124:927–34. Epub 2015/11/13. doi: 10.1289/ehp.1409529 . 26566198 PMC4937850

[74] Son J-Y, Lane KJ, Lee J-T, Bell ML. Urban vegetation and heat-related mortality in Seoul, Korea. Environ Res. 2016; 151:728–33. Epub 2016/09/17. doi: 10.1016/j.envres.2016.09.001 . 27644031 PMC5071166

[75] Tieges Z, McGregor D, Georgiou M, Smith N, Saunders J, Millar R, et al. The Impact of Regeneration and Climate Adaptations of Urban Green-Blue Assets on All-Cause Mortality: A 17-Year Longitudinal Study. Int J Environ Res Public Health. 2020; 17. Epub 2020/06/25. doi: 10.3390/ijerph17124577 . 32630538 PMC7344529

[76] Federal Statistical Office Germany. Krankenhäuser: Kreise, Krankenhäuser, Betten, Patienten, Ärzte, Pflegepersonal, Jahr. © Statistisches Bundesamt (Destatis) [updated 8 Feb 2021; cited 8 Feb 2021]. https://www.statistikdaten.bayern.de/genesis//online/data?operation=table&code=23111-001r.

