# Supplementary figures and images for: Universal thermal climate index associations with mortality, hospital admissions, and road accidents in Bavaria
Source: PLoS One. 2021 Nov 17;16(11):e0259086. doi: 10.1371/journal.pone.0259086 (PMC8598056; doi:10.1371/journal.pone.0259086)

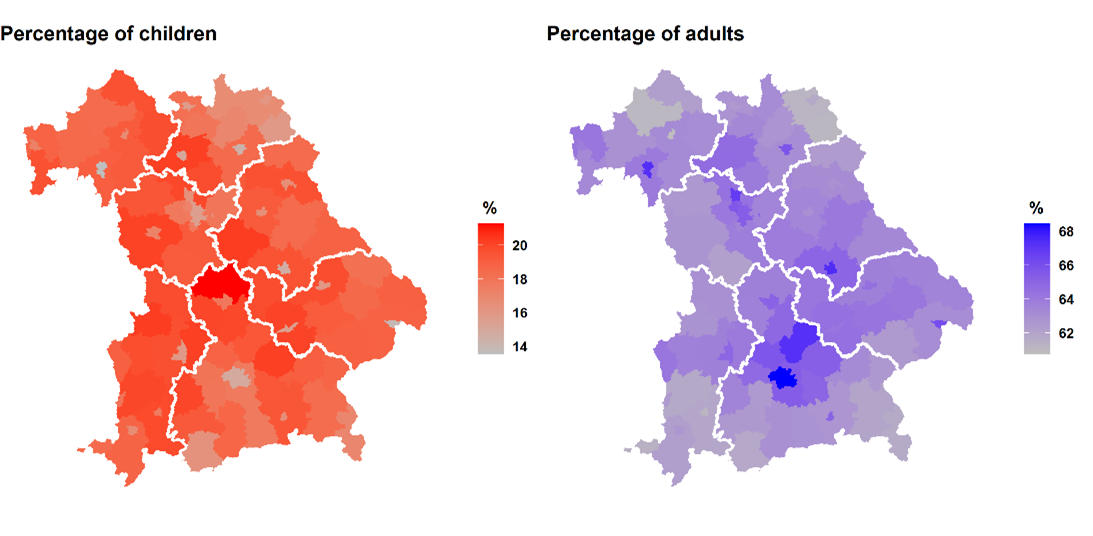

Supplement: S1 Fig — The percentage of children and adults. All values are averaged over the study period 1995–2015 [55]. Border shapefiles were provided with written permission by the Bundesamt für Kartographie und Geodäsie under the license (CC BY 4.0). (TIF) [file pone.0259086.s001.tif]

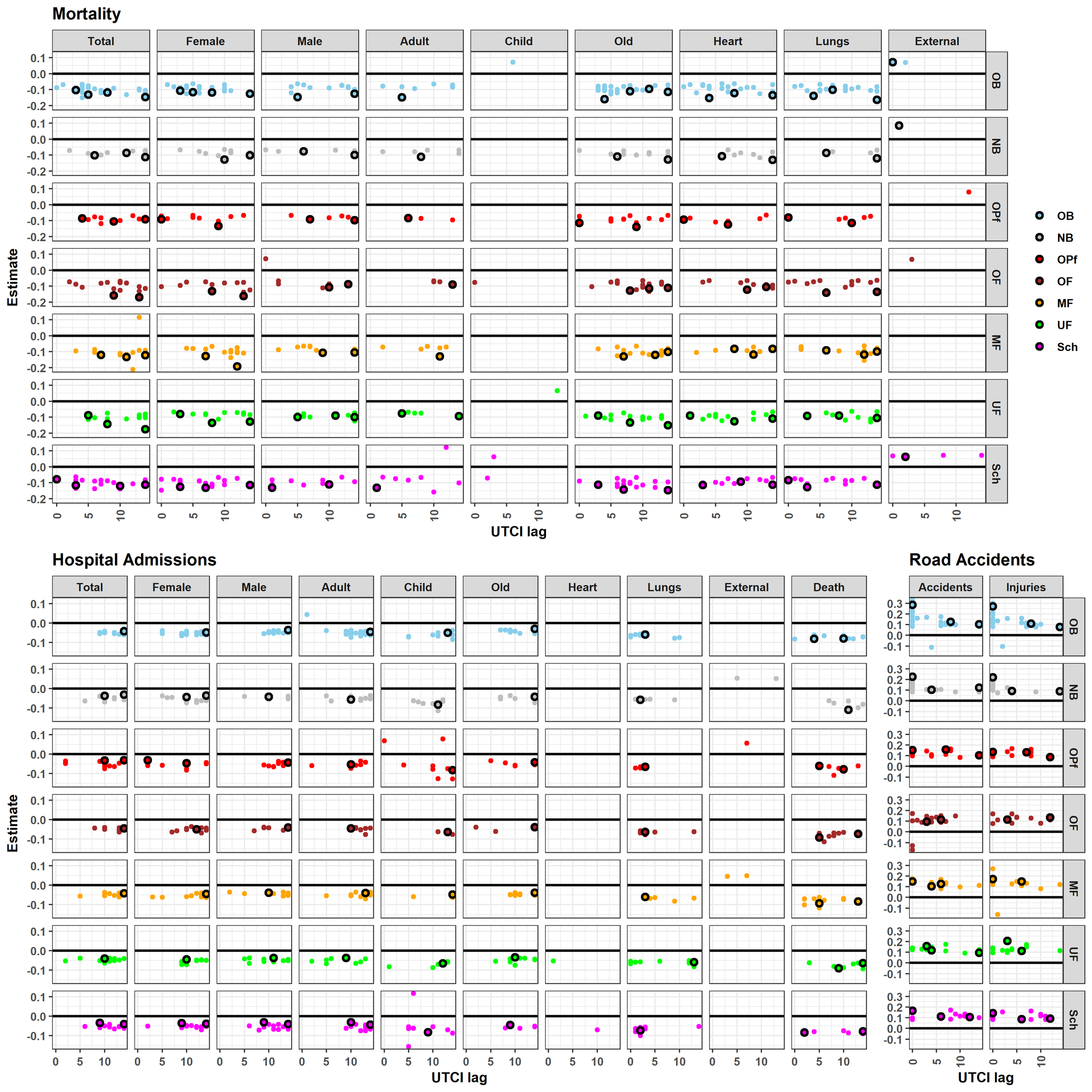

Supplement: S2 Fig — Spring UTCI effect on the daily mortality, hospital admissions, and road accidents for each subgroup. The horizontal axis represents the lag in days, and the vertical axis represents the effect estimate. It is expressed in proportion of the standard deviations of daily number of cases within the corresponding subgroup when UTCI changes by one standard deviation of its daily value. The black circles represent those effects for each region. The colored dots represent those effects for districts within the region. The absence of the points means that the effect of UTCI was not significant for a particular lag in the corresponding model. (TIF) [file pone.0259086.s002.tif]

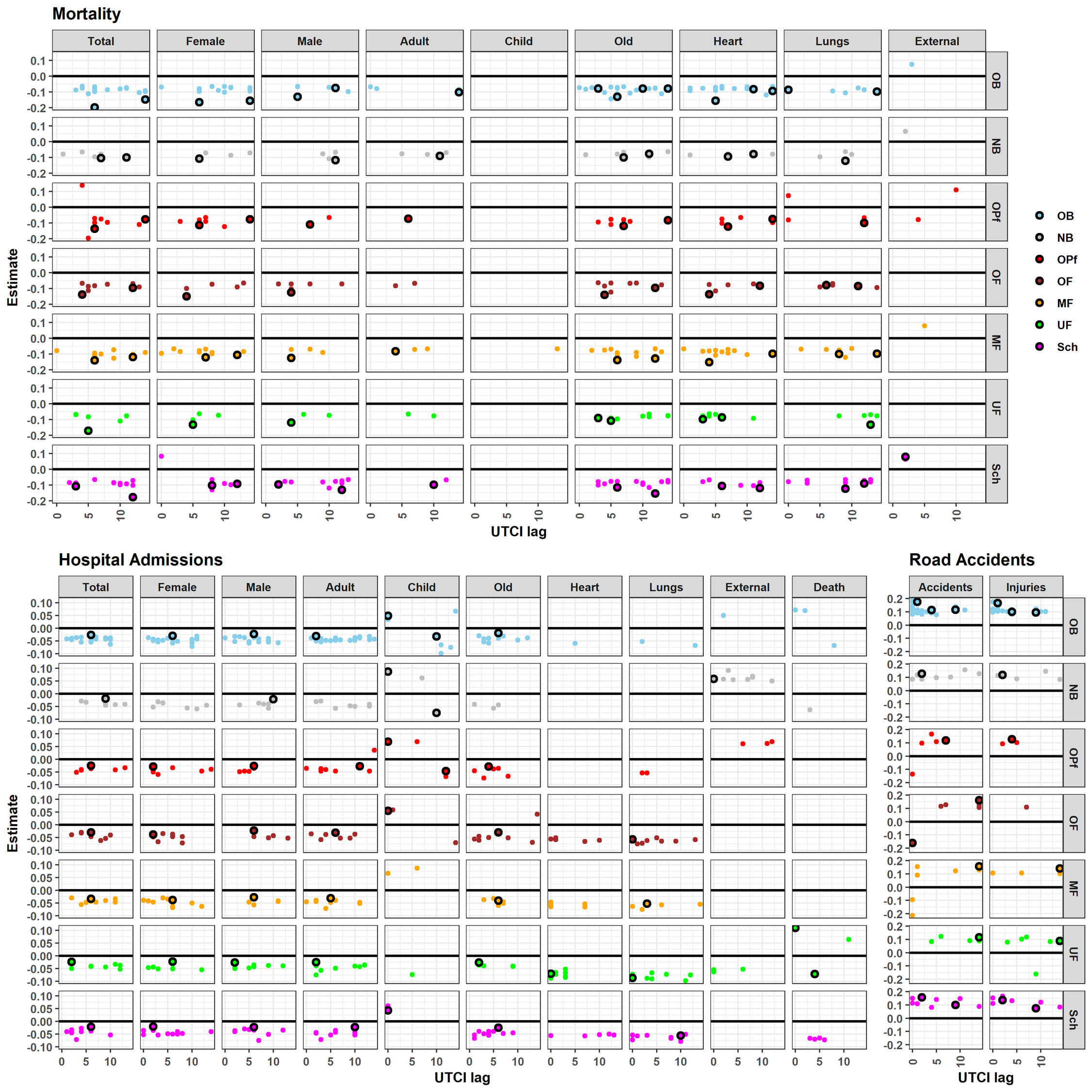

Supplement: S3 Fig — Fall UTCI effect on the daily mortality, hospital admissions, and road accidents for each subgroup. The horizontal axis represents the lag in days, and the vertical axis represents the effect estimate. It is expressed in proportion of the standard deviations of daily number of cases within the corresponding subgroup when UTCI changes by one standard deviation of its daily value. The black circles represent those effects for each region. The colored dots represent those effects for districts within the region. The absence of the points means that the effect of UTCI was not significant for a particular lag in the corresponding model. (TIF) [file pone.0259086.s003.tif]

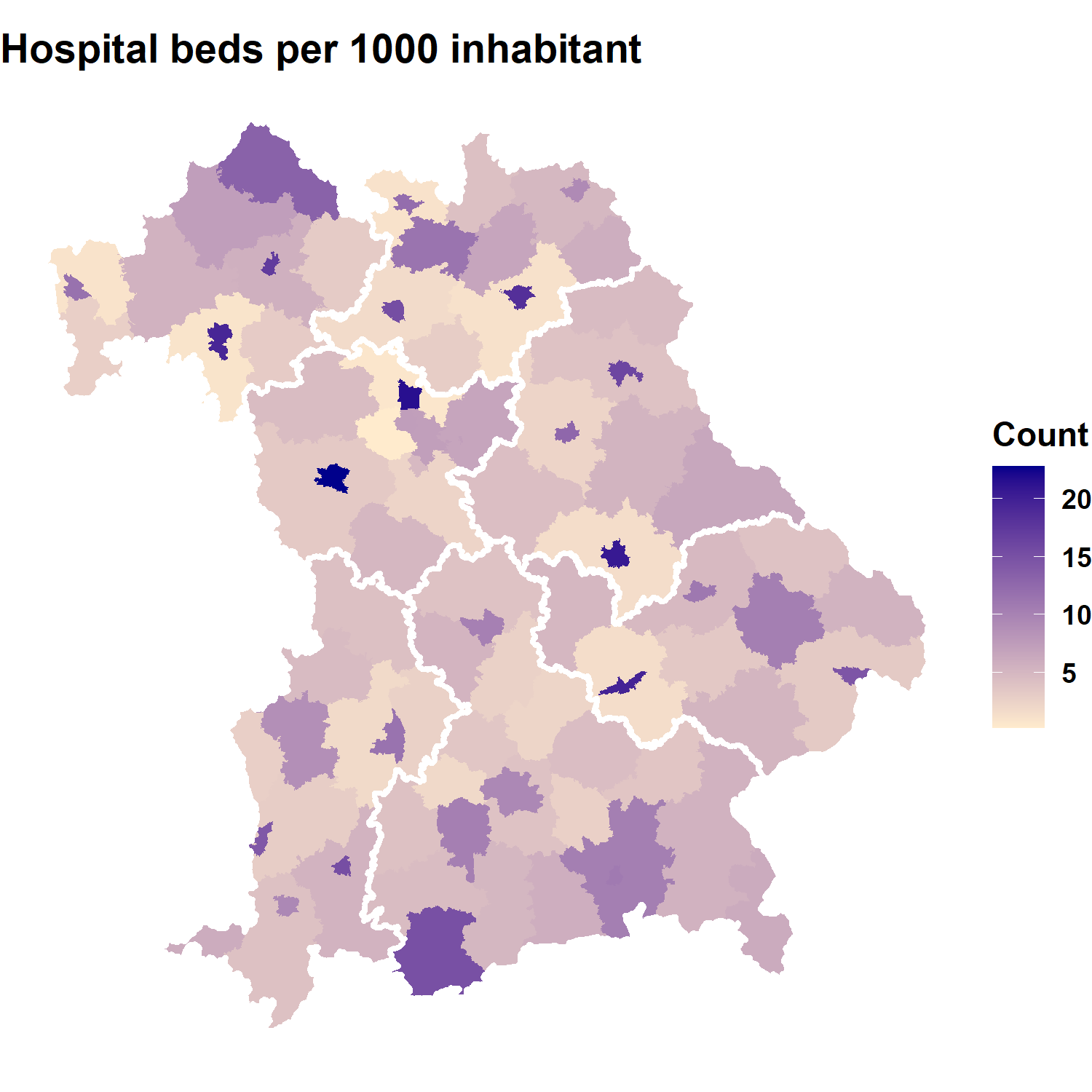

Supplement: S4 Fig — The number of hospital beds per 1000 inhabitants averaged over the study period 1995–2015 [76]. Border shapefiles were provided with written permission by the Bundesamt für Kartographie und Geodäsie under the license (CC BY 4.0). (TIF) [file pone.0259086.s004.tif]
