# Supplementary material for: Universal thermal climate index associations with mortality, hospital admissions, and road accidents in Bavaria
Source: PLoS One. 2021 Nov 17;16(11):e0259086. doi: 10.1371/journal.pone.0259086 (PMC8598056; doi:10.1371/journal.pone.0259086)
Supplement: S1 Table — (DOCX) [file pone.0259086.s005.docx]

| Regions | | Niederbayern  (NB) | Oberbayern  (OB) | Oberfranken  (OF) | Oberpfalz  (OPf) | Schwaben  (sCH) | Unterfranken  (UF) | Mittelfranken  (MF) |
| --- | --- | --- | --- | --- | --- | --- | --- | --- |
| Hospital admissions (1995-2015) | | | | | | | | |
| Total | | 5168521 | 19737933 | 4789391 | 5127236 | 7174579 | 6179137 | 7851571 |
| Distribution among subgroups % | Female | 54.06 | 54.1 | 53.9 | 52.6 | 54.2 | 52.6 | 53.3 |
|  | Male | 45.9 | 45.9 | 46.1 | 47.5 | 45.8 | 47.4 | 46.7 |
|  | Adult | 57.2 | 59.6 | 56.7 | 58.6 | 56.9 | 59.2 | 58.4 |
|  | Child | 10.9 | 10.6 | 9.7 | 10.7 | 11.6 | 11.5 | 11.3 |
|  | Old | 32.0 | 29.8 | 33.6 | 30.6 | 31.5 | 29.2 | 30.3 |
|  | Heart | 3.7 | 3.4 | 3.9 | 3.6 | 3.4 | 3.7 | 3.4 |
|  | Lungs | 1.7 | 1.2 | 1.4 | 1.6 | 1.7 | 1.4 | 1.6 |
|  | External | 2.8 | 2.4 | 2.5 | 2.6 | 2.7 | 2.6 | 2.4 |
|  | Death | 2.3 | 2.0 | 2.6 | 2.4 | 2.1 | 2.0 | 2.1 |
| MORTALITY (1995-2015) | | | | | | | | |
| Total | | 250326 | 799492 | 261829 | 229686 | 370202 | 276525 | 369591 |
| Distribution among subgroups % | Female | 52.4 | 53.6 | 52.8 | 52.2 | 53.4 | 52.6 | 53.1 |
|  | Male | 47.7 | 46.4 | 47.2 | 47.9 | 46.6 | 47.4 | 46.9 |
|  | Adult | 38.2 | 37.2 | 38.8 | 39.5 | 36.5 | 36.7 | 38.6 |
|  | Child | 0.7 | 0.6 | 0.5 | 0.7 | 0.7 | 0.6 | 0.6 |
|  | Old | 61.1 | 62.2 | 60.7 | 59.9 | 62.8 | 62.7 | 60.8 |
|  | Heart | 45.2 | 42.9 | 48.0 | 45.3 | 45.3 | 46.4 | 45.8 |
|  | Lungs | 6.8 | 6.6 | 6.8 | 6.8 | 6.4 | 6.5 | 7.1 |
|  | External | 4.2 | 4.3 | 3.5 | 4.0 | 4.1 | 3.6 | 3.7 |
| Road accidents (2002-2015) | | | | | | | | |
| Total | | 87132 | 325471 | 83788 | 81945 | 133914 | 92294 | 126317 |
| Distribution among regions % | | 9.4 | 35.0 | 9.0 | 8.8 | 14.4 | 9.9 | 13.6 |
| Injured persons (2002-2015) | | | | | | | | |
| Total | | 159526 | 627360 | 151914 | 151628 | 250400 | 171573 | 241579 |
| Distribution among regions % | | 9.1 | 35.8 | 8.7 | 8.6 | 14.3 | 9.8 | 13.8 |
